# Individual Differences in Emotion Attenuation Brought by Indirect Replies Is Related to Resting-State Brain Activity

**DOI:** 10.3390/brainsci13071053

**Published:** 2023-07-10

**Authors:** Xiuping Zhang, Maoyao Xu, Xiaohong Yang, Yufang Yang

**Affiliations:** 1 School of Psychology, Beijing Language and Culture University, Beijing 100083, China; zhangxp@blcu.edu.cn (X.Z.); 202221198655@stu.blcu.edu.cn (M.X.); 2 Department of Psychology, Renmin University of China, Beijing 100872, China; 3 CAS Key Laboratory of Behavioral Science, Institute of Psychology, Beijing 100101, China; 4 Department of Psychology, University of Chinese Academy of Sciences, Beijing 100049, China

**Keywords:** indirect replies, emotional experience, rs-fMRI, left caudate, rACC, rACC-mPFC

## Abstract

During daily conversations, people prefer indirect replies in face-threatening situations. Existent studies have indicated that recipients tend to perceive the information conveyed by indirect replies as negative and emotion regions are engaged in indirect replies processing in face-threatening situations. In this study, we examined whether indirect replies can reduce recipients’ experience of negative emotion and what are the underlying cerebral structures that may give rise to individual differences in the effectiveness of such replies in attenuating negative emotion. Behavior ratings and resting-stating functional magnetic resonance imaging (rs-fMRI) techniques were combined to explore these questions. We created dialogues expressing refusal or negative opinion with direct/indirect replies. Participants were asked to rate their emotional valence and arousal when they received such replies. The rating scores were used to correlate with spontaneous brain activity. Results showed that indirect replies indeed attenuated recipients’ negative emotion experience. Moreover, the left caudate, the right anterior cingulate cortex (rACC), and the connectivity of rACC and left medial prefrontal cortex (lmPFC) were found to be positively correlated to individual differences in such emotion attenuation. Our findings provide direct empirical evidence for the face-saving function of indirect replies and reveal that the intrinsic brain activities of emotion network and theory of mind (ToM) network are related to individual differences in such emotion attenuation.

## 1. Introduction

Imagine that a student asks his teacher, “Do you like my presentation?” The teacher may respond by stating “It is hard to give a good presentation”. In this dialogue, the teacher does not give a direct answer but successfully conveys the information that he does not like his presentation. The reply in this dialogue is an utterance that appears to be irrelevant to the student’s question. However, the student might understand that disapproving message is strategically conveyed in order to save his face. Such indirect replies are very common in daily conversation, especially in potentially face-threatening situations, in which refusal, negative opinion or negative self-disclosure would be expressed [1,2,3]. The preferences for indirect replies could be accounted for by the Politeness Theory, which assumes that maintaining one’s own face and an interlocutor’s face is an important aspect of cooperative behavior [4,5].

Numerous experimental studies have demonstrated readers’ sensitivity to the face-saving function of indirect replies [1,2]. To figure out the intended meanings of indirect replies, comprehenders typically recruit language processing regions and additionally, the theory of mind (ToM) network, including the bilateral inferior frontal gyrus (IFG), bilateral middle temporal gyrus (MTG), medial prefrontal cortex (mPFC), temporal–parietal junction (TPJ) and precuneus [6,7]. These brain regions are related to the detection of contextual relevance as well as the generation of inferences pertaining to the speakers’ implicit message [8,9,10,11,12,13].

Moreover, compared to informative–indirect replies, face-saving indirect replies induced enhanced activity in emotion-related areas, including the right anterior insula, ACC, and the caudate, the regions implicated in the processing of emotional salience [8,12,13,14,15] and emotion regulation [16,17,18,19,20]. Particularly, one meta-analysis study on emotion regulation [17] has established that the anterior middle cingulate cortex may play a central, integrative role in emotion regulation. The involvement of these emotion-related areas in the processing of face-saving indirect replies implies that readers could infer the negative attitude/opinion (such as refusal) conveyed by indirect replies and recognize speakers’ kind intentions to save the recipients’ face and show politeness by using indirect replies [8].

As aforementioned, previous studies have indicated that in face-threatening situations, speakers tend to convey negative information via the use of indirect replies so as to save recipients’ face. Despite this, it remains unclear how the use of indirect replies affects the emotional experience of the recipient. According to the Politeness Theory [4,5], indirect replies are more socially acceptable for the recipients when it comes to the expression of negative opinions or refusals. Considering this, it is plausible to assume that the usage of indirect replies may result in a reduced negative emotional response from the recipient. However, this remains to be tested. Thus, in the present study, the first question we examined is whether indirect replies can attenuate recipients’ negative emotion experience.

Moreover, several studies have identified variations in the comprehension of indirect language amongst individuals, including working memory capacity [21], intercultural differences [22,23], gender differences [24] and differences in language-use style [22]. Holtgraves (1997), for instance, found great differences in the interpretation of conversational indirectness among people, with some individuals tending to search for hidden meanings in others’ remarks, whereas others usually interpret remarks in a literal manner. Given that the interpretation of others’ remarks varies amongst individuals, the impact of indirect replies on individuals’ emotional experiences may similarly differ.

If individuals do differ in their emotional experience when they receive indirect replies, they may have different functionally relevant intrinsic brain activity. It is well established that spontaneous brain activity may encode or support the encoding of behaviorally relevant information [25]. The strength of correlation between spontaneous brain activity and behavior information can be associated with individual differences in cognitive processes. Thus, if indirect replies can reduce negative emotional experiences, an interesting question follows naturally: what is the underlying cerebral structure that may give rise to individual differences in the attenuation of emotions?

In order to examine whether indirect replies attenuate recipients’ negative emotion experience in face-threatening situations, we created dialogues expressing refusal or negative opinion with direct/indirect replies in face-threatening situations. Participants were asked to rate the emotional valence and arousal when they received such replies, through which we can directly compare the difference of emotional experience between direct and indirect replies. Moreover, in order to explore the underlying cerebral structure that may give rise to individual differences in emotion attenuation, we combined behavior ratings with resting-state functional magnetic resonance imaging (rs-fMRI).

Rs-fMRI is a promising way to explore the relationship between individuals’ spontaneous brain activity and their behavior performance. Similar to the task-based fMRI method, the correlation of behavior performance in language processing and resting-state brain activity has been found to be an effective measure of the neural mechanism underlying language comprehension. Several studies have found that the brain region and network identified by the correlation of behavior performance and resting-state brain activity overlapped with those observed by task-based functional brain-imaging studies [26,27,28,29].

Of the rs-fMRI measures, the amplitude of low-frequency fluctuations (ALFF) and fractional ALFF (fALFF) and functional connectivity (FC) analyses have been extensively used [30,31,32]. ALFF [30] and fALFF [31] reflect the intensity of local brain activity as local indicators while FC reflects the strength of functional connectivity between brain regions [33]. Healthy individuals’ resting-state ALFF/fALFF and resting-state FC have been reliably found to correlate with task-evoked BOLD responses and participants’ behavioral measures [27], such as semantic processing [28], word-reading skill [29], individuals’ reading ability [34], phonological processing [35], and language preoperative planning [26]. Given the robustness of these measures, in the present study we investigated whether indirect replies could attenuate recipients’ negative emotional experience and if so, whether their spontaneous brain activity is related to individual variations in emotional reduction, using ALFF/fALFF and FC of resting state fMRI signals.

According to the Politeness Theory [4,5], we hypothesized that indirect replies might be more polite than direct replies to manage the recipients’ face and thus indirect replies would attenuate the recipients’ negative experience. On the basis of previous studies [8,14,15], we predicted that emotion-related areas, such as ACC/Insula/caudate, might be related to the emotional rating scores. In order to understand indirect replies, in particular to understand speakers’ intentions, it is necessary for readers to think about speakers’ mental states to infer their intended meanings [8,15]. Thus, brain regions involved in ToM processing, such as mPFC/TPJ [8,9,10,11,12,13,15], might also be related to negative emotion attenuation together with emotional brain areas.

## 2. Methods

### 2.1. Ethics Statement

All participants signed written informed consent in accordance with the Declaration of Helsinki. The ethics committee of the Institute of Psychology, Chinese Academy of Sciences, approved this study, its participant recruitment procedure and its methodology.

### 2.2. Participants

Fifty-three undergraduate and graduate students participated in the experiment (25 males, mean age = 23.2 years, range = 19–28 years). All were right-handed native Mandarin Chinese speakers with normal or correct-to-normal vision. They reported no reading problems, neurological impairment or psychiatric disorder. All participants were paid for their participation.

### 2.3. Materials

Seventy sets of scenarios were selected by conducting two pretests (see below). There were two scenarios in each set that shared the same contexts but had different critical replies (see Table 1). Each scenario comprised a cover story which briefly introduced the communication circumstance, a yes/no question, and a direct/indirect reply to the question. There were two different conditions in the experimental design: direct reply (DI) and indirect reply (IN). All the scenarios contained *You* (to guide the participants to imagine as themselves) and a character (e.g., *Zhang Zheng)* who gave replies to the question asked by *You*. All the conversations conveyed information about refusal or negative opinion via direct/indirect replies.

The 70 sets of scenarios were divided into two versions using a Latin-square design to ensure that each scenario set was only presented once per version. Each version contained 35 discourses per condition. To each version, 60 fillers were added which had similar structures with the two experimental conditions (30 dialogues with positive emotion and 30 neutral dialogues, both with direct and indirect replies). These fillers were used to balance emotional valence.

#### Pretests of Materials

A pretest was conducted to assess the level of indirectness of the dialogues in order to select the experimental stimuli. Twenty participants who did not participate in the formal experiment took part in the indirectness pretest. We generated 90 sets of scenarios and divided them into two lists according to a Latin-square procedure. Each list was rated by 10 participants. During this pretest, participants were asked to read carefully and rate how directly the reply answered the question on a 7-point scale (1 representing the most direct reply and 7 representing the most indirect reply). If the reply was indirect (5–7), participants were asked to fill in the intended meaning conveyed by the speaker. We encoded what participants filled in as 0 (not understanding what the speaker meant) and 1 (correctly understanding what the speaker meant). The scenarios were selected according to criteria as follows: ratings for direct reply ≤ 2, ratings for indirect reply ≥ 4.5, and comprehension accuracy > 75%. Thus, seventy sets of scenarios were selected for the formal experiment. A paired T test showed that the indirectness level of an indirect reply (*M* = 5.55, *SD* = 0.32) was significantly higher (*t*_(69)_ = 315, *p* < 0.001) than that of a direct reply (*M* = 1.35, *SD* = 0.28). The mean comprehension accuracy for an indirect reply was 90% (*SD* = 10%).

### 2.4. Procedure

#### 2.4.1. Behavioral Test

**Figure 1:**
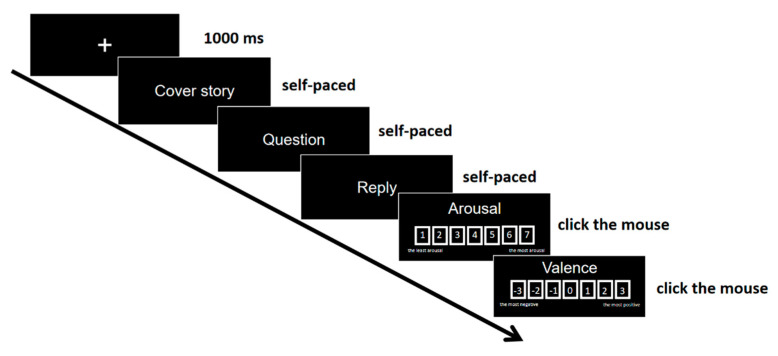
The procedure of the behavioral test.

Participants sat in a comfortable chair in a sound-attenuating shielded room and were instructed to read each scenario carefully for comprehension. The scenarios were presented visually in the center of the computer screen. The procedure of the behavioral test is shown in Figure 1. Each trial started with a fixation cross (+) in the center of the screen for 1000 ms. Then, the cover story appeared in the screen. Participants were asked to press the space bar when they finished reading the cover story. After that, the question asked by *You* (we asked participants to imagine *You* as themselves) was presented in the center of the screen till participants pressed the space bar and the replies were presented in the center of the screen. After they read the replies and pressed the space bar, the participants were asked to rate their own emotional arousal (1 to 7, 1 representing the least arousal and 7 representing the most arousal) and valence (−3 to 3, −3 representing the most negative and 3 representing the most positive) brought by the replies in each scenario by clicking the mouse. Participants were required to involve themselves as much as possible into the scenarios and to rate their emotional experience as if they had received such replies.

The stimuli were divided into three blocks. Each block lasted about 12 min and there was a short break between blocks. The discourses were presented in a pseudo-random order and no more than three scenarios for the same condition were presented in succession. Before the formal experiment, a practice of six trials was conducted to familiarize participants with the procedure.

#### 2.4.2. Image Acquisition and Data Preprocessing

Images were acquired using a GE Discovery MR750 3T scanner at Magnetic Resonance Imaging Research Center, Institute of Psychology, CAS. The manufacturer of the equipment is GE Healthcare, located in Chicago, IL, USA. The participants lay supine and their heads were fixed with straps and foam pads in order to minimize head motion and scanner noise. Functional images were collected using an echoplanar imaging sequence with the following parameters: TR = 2000 ms, TE = 30 ms, flip angle = 70°, matrix size = 64 × 64. For 16 participants, 33 slices, slice thickness = 3.5 mm, FOV = 224 mm × 224 mm, voxel size = 3.5 mm × 3.5 mm × 4.2 mm; for 25 participants, 35 slices, slice thickness = 3.5 mm, FOV = 220 mm × 220 mm, voxel size = 3.4 mm × 3.4 mm × 4 mm; for 20 participants, 37 slices, slice thickness = 3.5 mm, FOV = 220 mm × 220 mm, voxel size = 3.4 mm × 3.4 mm × 4 mm. T1-weighted structural images were collected in 176 sagittal slices with 1.0 mm isotropic voxels. During rs-fMRI scanning, participants were asked to stare at the screen with a fixation dot, keep still, and not think about anything systematically or fall asleep. The scanning for rs-fMRI lasted for eight minutes (240 time points). Since the MRI Research Center of the IPCAS had its own mandatory scanning parameters for the acquisition of resting-state fMRI data, the slice number was slightly changed during the performance of our study.

Preprocessing of the resting fMRI data was performed using the advanced edition of DPARSF V4.3 [36] (http://www.restfmri.net, accessed on 1 March 2020), which is based on SPM12 software (http://www.fil.ion.ucl.ac.uk/spm, accessed on 13 January 2020) and the toolbox for Data Processing and Analysis of Brain Imaging (DPABI_V5.0, [37], http://rfmri.org/DPABI, accessed on 1 October 2020). First, the initial 10 volumes were discarded for steady-state magnetization, and slice-timing correction was performed separately for the three groups (different scanning slices). Except for slice-timing correction, all subsequent preprocessing was performed with all participants together. After slice-timing, realignment was performed using a six-parameter (rigid body) linear transformation. Subsequently, T1-weighted images were co-registered to the mean functional image and then segmented into gray matter, white matter, and cerebrospinal fluid [38]. Then, the images for each subject were transformed from individual native space to MNI space [39], and spatially smoothed with a Gaussian kernel (FWHM = 4 mm). After that, the linear trend was removed and temporal band-pass filtering (0.01–0.1 Hz) was applied only for FC analysis to reduce low-frequency drift and physiological high-frequency noise.

Participants whose head motion was greater than 3° in rotation or 3 mm in any direction throughout the resting state fMRI scan were excluded from further analyses. Two participants (one female) who met this criterion were excluded, and 53 participants were left for further analyses. In addition, the mean framewise displacement (FD) [40] was used as a covariate in the statistical analysis to exclude confounding effects of head motion.

### 2.5. Data Analysis

#### 2.5.1. Behavioral Data Analysis

The arousal (1 to 7) and valence (−3 to 3) scores for direct and indirect replies were averaged separately. Then, we used the valence scores and arousal scores of indirect replies to subtract those of direct replies respectively, and we used their absolute values as the valence and arousal difference. In such a way, the valence difference and the arousal difference are both positively related to emotion attenuation, i.e., the greater the difference, the greater extent of emotion attenuation brought by indirect replies. Then, the valence and arousal difference scores were used to correlate with the spontaneous brain activity, respectively.

#### 2.5.2. ALFF/fALFF Calculation and ALFF/fALFF-Behavior Analysis Procedures

For resting-state activity, we used DPASF (http://www.restfmri.net, accessed on 1 March 2020) to calculate voxel-wise ALFF/fALFF for each subject. ALFF and fALFF quantify local resting-state signal fluctuations and index fluctuations within the low-frequency range (0.01–0.1 Hz). ALFF is calculated as the sum of the signal amplitude within the low-frequency range (0.01–0.1 Hz), and fALFF represents the fluctuations within the low-frequency range, relative to the fluctuations within the whole detectable frequency range. In other words, fALFF is a normalization of ALFF with respect to all detectable frequencies in the measured signal. Participant-level voxel-wise ALFF/fALFF maps were converted into Z-score maps by subtracting at each voxel the mean ALFF/fALFF obtained for the whole brain and dividing by its standard deviation [41]. Then, we correlated the difference of arousal and valence between indirect reply and direct reply to Z-score maps of ALFF/fALFF among participants, and the GRF correction (z > 2.3, voxel *p* < 0.01; cluster *p* < 0.05) was used for multiple comparison [35,42]. For the ALFF/fALFF-behavior correlation analysis, four variables were controlled as the covariates. The first was the slice number. Since the scanning parameters among the participants were different, a binary variable (10 for 33 slices, 01 for 35 slices and 00 for 37 slices) was set as a logical covariate to control its effects. In order to control the effects of head motion, we used the mean FD_Jenkinson [43] to represent the head motion measure [44]. The other two covariates were gender (a binary variable, 0 for females and 1 for males) and age (a continuous variable).

#### 2.5.3. Functional Connectivity (FC) Analysis and FC-Behavior Analysis Procedures

Functional connectivity (FC) analysis was performed using the DPASF (http://www.restfmri.net, accessed on 1 March 2020). Two seeds identified in the ALFF/fALFF-behavior correlation analysis (i.e., the left caudate and the right ACC) were used for FC analysis. In order to obtain the functional connectivity map for each participant, firstly, we calculated the mean time series of the seeds for each participant. Then, the correlation coefficients (*r*) between the seeds time series and other voxels was calculated to obtain an *r* map for each participant. Fisher *z* score transformations were performed for the correlation coefficients (*r*) to obtain a z-FC map for each participant. After that, we examined whether any specific connections within this network are related to individual variations of emotion attenuation. Specifically, correlation analyses between each participant’s emotional rating scores and their z-FC maps were performed, and the GRF correction (z > 2.58, voxel *p* < 0.005; cluster *p* < 0.05) was used for multiple comparisons [35,42,45]. Similar to the ALFF/fALFF-behavior correlation analysis, four variables were also controlled as the covariates for the FC-behavior correlation analyses: slice number, head motion, gender and age.

## 3. Results

### 3.1. Behavioral Results

**Figure 2:**
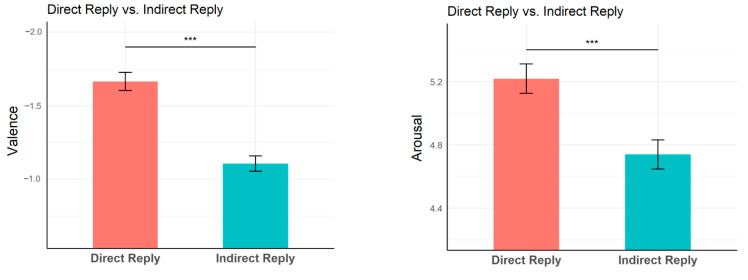
Valence rating scores (−3~3, left panel) and arousal rating scores (1~7, right panel) for target sentences in each condition (indirect reply and direct reply). Error bars represent the standard error. *** represents *p* < 0.001.

The valence results (left panel) and the arousal results (right panel) were shown in Figure 2. The valence of indirect reply (*M* = −1.11, *SD* = 0.05) was significantly less negative ([*t*_(52)_ = 14.0, *p* < 0.001]) than that of direct reply (*M* = −1.67, *SD* = 0.06). The arousal of indirect reply (*M* = 4.74, *SD* = 0.09) was significant lower [*t*_(52)_ = 11.3, *p* < 0.001] than that of direct reply (*M* = 5.22, *SD* = 0.09). These results suggest that indirect replies indeed attenuated negative emotional experience in face-threatening situations.

### 3.2. Regional ALFF/fALFF Analysis and ALFF/fALFF-Behavior Analysis

**Figure 3:**
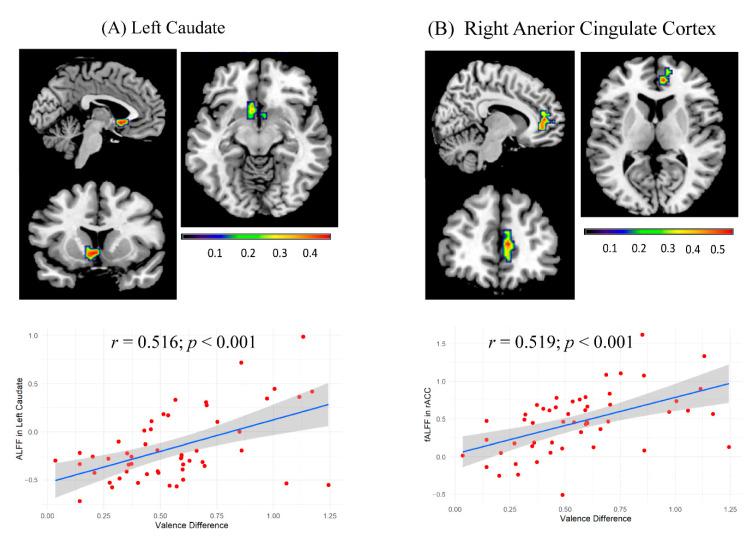
Correlations of valence difference (indirect replies–direct replies) and ALFF/fALFF values. Significant statistical maps for the correlations of valence difference and ALFF/fALFF values, including (**A**) left caudate (peak: −3, 12, −6; 114 voxels) (**B**) right anterior cingulate cortex, rACC (peak: 9, 45, 9; 63 voxels). Scatter plots show the correlations of valence difference and ALFF values in left caudate (*r* = 0.516; *p* < 0.001) and fALFF values in right ACC (*r* = 0.519; *p* < 0.001). Each red dot represents valence difference data from one participant, with 53 participants in total. The blue line represents the trend line in scatter plots.

To explore the association between emotion attenuation and regional resting-state activity, we first correlated the arousal/valence difference with the ALFF/fALFF value of each voxel across the whole brain. The ALFF of one region, the left caudate [peak, −3, 12, −6; 114 voxels; Figure 3A], was positively correlated with participants’ valence difference between indirect and direct replies (*r* = 0.516; *p* < 0.001, see Figure 3A). The fALFF of one region, the right ACC [peak, 9, 45, 9; 63 voxels; Figure 3B], was also positively correlated with participants’ valence difference between indirect and direct replies (*r* = 0.519; *p* < 0.001, see Figure 3B). No significant correlation was found between arousal difference and regional spontaneous brain activity.

### 3.3. Functional Connectivity Analysis and Connectivity–Behavior Analysis

**Figure 4:**
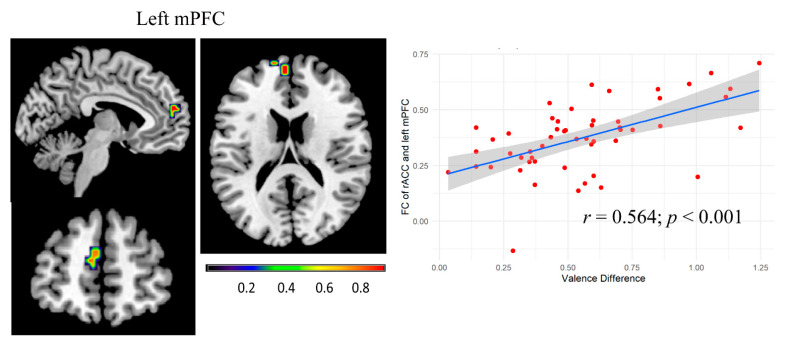
Correlation of valence difference (indirect replies–direct replies) and the functional connectivity of rACC and left mPFC (peak: −6, 54, 18; 26 voxels). Scatter plot shows the correlation of valence difference and FC strength of rACC and left mPFC (*r* = 0.564; *p* < 0.001). Each red dot represents valence difference data from one participant, with 53 participants in total. The blue line represents the trend line in scatter plots.

To explore whether the left caudate and the right ACC function in concert with other brain regions for emotion attenuation, we performed a seed voxel correlation analysis between the left caudate, the right ACC region and all other voxels in the brain. Results showed that the strength of functional connectivity between the right ACC and the left medial prefrontal cortex (mPFC) was positively correlated with valence difference scores [peak, −6, 54, 18; 26 voxels; *r* = 0.564, *p* < 0.001; Figure 4]. The strength of functional connectivity between the left caudate and other brain regions was not significantly correlated with valence difference scores.

## 4. Discussion

In the present study, we firstly examined whether face-saving indirect replies could attenuate recipients’ negative emotion experience compared to non-face saving direct replies. Additionally, we used the correlation between behavior ratings and resting-state fMRI to further explore the relationship between spontaneous brain activity and individual variations of emotion reduction induced by indirect replies. The behavior results showed that compared to direct replies, indirect replies were perceived as less negative in valence and less emotionally aroused. As for the behavior-rfMRI correlation analysis, we found that emotion attenuation brought by indirect replies (valence difference scores) was positively correlated with the amplitude of ALFF activity in left caudate and the amplitude of fALFF in right ACC. In addition, the FC of right ACC and left mPFC were positively related to emotion attenuation. These results suggest that in face-threatening situations, indirect replies can attenuate recipients’ negative emotion experience. Moreover, individual differences in the extent to which negative emotions are reduced by indirect responses might be related to the intrinsic activity of emotional areas and the functional connectivity of the emotional area with the ToM processing area.

### 4.1. Face-Saving Indirect Replies Attenuate Recipients’ Negative Emotion Experience

Our behavior results showed that in face-threatening situations, indirect replies could attenuate recipients’ negative emotion experience. These results provide empirical support for the hypothesis that indirect replies function as face management in face-threatening situations [1]. According to the politeness theory [3,4], indirectness is an important approach to convey politeness, and politeness is motivated by face management [1]. Thus, in the present study, the observation that individuals attenuate negative emotions more readily when exposed to indirect rather than direct replies indicates that the former may be employed as a more socially acceptable means of communicating negative information, thus allowing the sender to better manage the recipients’ face.

### 4.2. The Intrinsic Brain Activity Underlying Individual Differences in the Attenuation of Negative Emotions Brought by Indirect Replies

The ALFF/fALFF results showed that individuals with stronger spontaneous activity of the left caudate and the right ACC experienced more reduction of negative emotion when they received indirect replies.

The caudate is one part of the ventral striatum, and a line of fMRI studies indicates that the ventral striatum is associated with emotion experience [46] and emotion regulation [47]. Similarly, many studies have associated ACC with emotional salience and emotional information processing [14,48,49,50,51,52,53]. As face-saving indirect replies express negative emotional information, some studies [8,15] have also found the involvement of ACC in the processing of face-saving indirect replies. Given that the caudate and the ACC are related to emotion processing and emotion attenuation, our results could be an indication that individuals with stronger spontaneous activity in the left caudate and the right ACC might be more sensitive to the emotional experience differences brought by direct replies and indirect replies, leading to more emotion attenuation brought by indirect replies.

In the present study, the left caudate showed a positive relationship with the valence difference only in the ALFF analysis and the right ACC only in fALFF analysis. This might be related to the fact that ALFF and fALFF are different measures of spontaneous brain activity which represent two levels of significance, and their retest reliability is also different [41]. In previous rfMRI studies, ALFF and fALFF were frequently found showing different results in the same set of data [32,54,55]. Given that ALFF and fALFF are different rfMRI measures, it is reasonable that different results were found for the two measures.

Interestingly, we also observed that individuals with stronger connectivity of right ACC and left mPFC tended to experience more reduction of negative emotions in response to indirect replies in face-threatening situations. Many studies have demonstrated that mPFC is a core region for Theory of Mind (ToM) processing [56,57,58,59,60,61,62,63,64] and intention processing [65]. Therefore, the observed correlation between mPFC-rACC functional connectivity and the valence difference scores may suggest that participants with stronger mPFC and rACC connectivity are more likely to infer the speakers’ mental state during conversation comprehension, e.g., why the speaker used indirect statements. As an indirect reply may function as face-management in face-threatening situations, it might be easier for participants with stronger mPFC-rACC functional connectivity to recognize the speakers’ kind intention to save the recipients’ face and to show politeness by using indirect replies. The degree of emotion attenuation resulting from an indirect reply may be closely related to the extent to which the speakers’ kind intention is comprehended. Consequently, greater comprehension of the speakers’ kind intention by participants is associated with a more significant reduction in negative emotions brought about by indirect replies.

Traditional pragmatic theories have posited two kinds of pragmatic processes, namely primary and secondary, which operate during utterance comprehension [66]. The primary pragmatic process is to understand what speakers say literally while the secondary pragmatic process is to understand what speakers have implied. In the present study, what the speakers have said was easily accessible for all the participants (as indicated by the comprehension accuracy in the pretest). However, those with stronger mPFC and rACC connectivity might be more capable of grasping what the speakers have intended, including their kindness in preserving the recipients’ dignity and showcasing their politeness [2,3]. As such, the mPFC might be related to the secondary pragmatic process to understand what speakers implicated [9,13,67]. Negative emotion attenuation can be deemed as the outcome of this process, as it involves recognizing the speakers’ kind intention. Thus, individuals with stronger connectivity of mPFC and rACC could experience more emotion attenuation brought by indirect replies.

One limitation of the present study is that we used a relatively lenient threshold. Based on previous rfMRI studies [35,42,45,54,68,69], we selected the present cluster-defining threshold. Although such a threshold has been extensively used in previous rfMRI studies [35,42,45,54,68,69], it should be noted that such a lenient threshold may increase familywise error rate. In future studies, a stricter threshold should be used to obtain more robust findings.

The other limitation is that the time resolution of the neuroimaging method makes it impossible to draw more accurate neurofunctional conclusions. In future studies, we will combine fMRI with EEG/MEG methods to reveal the neural processes of emotion attenuation more clearly towards more accurate conclusions.

## 5. Conclusions

In conclusion, compared to direct replies, indirect replies indeed attenuate participants’ negative emotion experience in face-threatening situations. Through behavior–rfMRI correlation, we observed that the left caudate, the right ACC and the connectivity of right ACC and left mPFC were positively correlated to individual differences in emotion attenuation brought by indirect replies. Our findings provide direct empirical evidence for the face-saving function of indirect replies and reveal that the intrinsic brain activity of the emotion network and the ToM network are related to individual differences in the reduction of negative emotion brought by indirect replies.

## Figures and Tables

**Table 1:** Example of experimental stimuli.

Condition	Cover Story	Dialogue	
	You are talking with Zhang Zheng, who is an acquaintance of yours. You hosted a party last week and Zhang Zheng attended the party. You decide to ask Zhang Zheng how he felt about the party.	**Question**	**Reply**
Indirect Reply		You: Did you enjoy yourself at my party?	Zhang Zheng: ***I prefer to be quiet.***
Direct Reply			Zhang Zheng: ***I didn’t have a good time.***

*Note.* The materials are originally in Chinese. The critical replies are in italics and bold. The word-by-word English translations are presented.

## Data Availability

The datasets generated for this study are available on request to the corresponding author.
